# Dietary magnesium intake as modifier of the association between vitamin D deficiency and risk of anemia in the US children aged 2–14 years: A cross-sectional study

**DOI:** 10.1097/MD.0000000000046264

**Published:** 2025-12-12

**Authors:** Wenxin Gu, Yue Wang, Chunhuai Li, Shuangshuang Wu, Shuangshuang Liu, Chun Chang, Lu Xue

**Affiliations:** a Department of Pediatrics Hematology, Children’s Medical Center, The First Hospital of Jilin University, Changchun, China.

**Keywords:** anemia, children, dietary magnesium intake, vitamin D deficiency

## Abstract

Vitamin D deficiency and magnesium deficiency are both associated with an increased risk of anemia. However, the impact of dietary magnesium intake on the relationship between vitamin D deficiency and anemia risk has yet to be determined. In this study, 11,471 children aged 2 to 14 years were enrolled using data from the National Health and Nutrition Examination Survey 2005 to 2018. The participants were divided into low and high magnesium intake groups based on a quantile cutoff of < 208.5 and ≥ 208.5 mg/d, respectively. Participants who were vitamin D deficiency had an increased risk of anemia (odds ratio = 1.492, 95% confidence intervals: 1.072–2.077). The incidence of anemia differed in low-dietary magnesium intake and high-dietary magnesium intake groups (odds ratio, 95% confidence interval: 1.992, 1.264–3.139 vs 0.934, 0.564–1.548, respectively), indicating an interaction between vitamin D deficiency and dietary magnesium intake in anemia risk (*P* = .009), which suggests that a magnesium-rich diet may reduce the risk of vitamin D deficiency-induced anemia. the findings highlight the synergistic effect of low-dietary magnesium intake and vitamin D deficiency on the increased risk of anemia in children.

## 1. Introduction

Vitamin D deficiency is a significant public health concern, especially considering the recent understanding of its clinical implications in conditions such as diabetes, osteoporosis, hypertension, cancer, and cardiovascular disease.^[1,2]^ An accumulating body of evidence suggests a close association between 25-hydroxyvitamin D (25(OH)D) deficiency and increased risk of anemia, as initially observed in adult studies^[3,4]^ and subsequently corroborated in pediatric populations.^[5,6]^ However, a small body of research has put forth differing viewpoints, with results indicating no association between 25(OH)D levels and anemia.^[7,8]^

In the United States, dietary magnesium intake frequently falls below recommended levels, resulting in approximately 28% of pregnant women experiencing anemia due to deficiency.^[9]^ Serum magnesium inversely correlates with anemia in both genders, especially in adults with high serum ferritin levels.^[10]^ Zeynep et al cross-sectional retrospective study revealed a positive relationship between magnesium deficiency and anemia in individuals with chronic kidney disease.^[11]^ Magnesium deficiency is closely linked to increased anemia occurrence, particularly in women and older Americans.^[12]^ Additionally, Cinar et al found that magnesium supplementation boosts hemoglobin levels in athletes.^[13]^

Magnesium is essential for various physiological processes and serves as a crucial cofactor for specific enzymes involved in vitamin D metabolism.^[14,15]^ All vitamin D-metabolizing enzymes appear to rely on magnesium as a cofactor, facilitating enzymatic reactions in the liver and kidneys.^[16]^ Both in vitro and in vivo studies have demonstrated the magnesium dependence of 1α-hydroxylase and 24-hydroxylase.^[14,15]^ However, limited clinical research exists on the relationship between vitamin D, magnesium intake, and the risk of anemia. Hence, this study aims to investigate the potential negative association between vitamin D deficiency and anemia in the general population, as well as the role of dietary magnesium intake in ameliorating this association.

## 2. Materials and methods

### 2.1. Ethics statement

The studies involving human participants were reviewed and approved by the Institutional Review Board of the National Center for Health Statistics (NCHS). The patients/participants provided their written informed consent to participate in this study.

### 2.2. Data sources and study population

The population and data were derived from the National Health and Nutrition Examination Survey (NHANES). They conducted large, multistage, stratified probability surveys to represent the noninstitutionalized population of US citizens. We used the NHANES public data continuously in our analysis from 2005 to 2018 (7 cycles in total: 2005–2006, 2007–2008, 2009–2010, 2011–2012, 2013–2014, 2015–2016, and 2017–2018). The original protocol was approved by the Ethics Review Board of the NCHS and can be accessed online (https://www.cdc.gov/nchs/nhanes/about/erb.html) www.cdc.gov/nchs/nhanes/irba98.htm. Written informed consent was obtained from all participants aged 18 years and above. For individuals below 18 years, consent was obtained from their guardians, and assent was obtained from those aged 12 to 17 years. The study was constructed through sequential exclusions as follows: *Inclusion criteria:* Aged 24 months to 14 years; Completed both interviews and examinations; Provided blood samples for CBC and vitamin D testing. *Exclusion criteria:* Age outside target range: <24 months or ≥15 years (n = 52,948); Missing core laboratory data: Hemoglobin or other CBC parameters included (n = 3627), serum 25(OH)D levels (n = 720); Missing dietary intake data (n = 1419) and abnormal data for dietary intake (n = 6). Ultimately, our study comprised a population of 11,471 US children. Other missing covariates included: missing values of 6.16% for poverty income ratio (PIR), 1.02% for household income, 5.84% for blood lead, and 1.51% for body mass index (BMI), all of which were <10%, and the data analysis was done using KNN interpolation.

### 2.3. Anemia ascertainment

Hemoglobin was determined with a Coulter Counter Model S-Plus JR (Beckman Coulter DxH 800 instrument, Brea, CA). Anemia was defined as hemoglobin values for anemia in children of different ages as prescribed by WHO.^[17]^ The World Health Organization defines anemia as hemoglobin level <11 g/dL in children 6 to 59 months of age, hemoglobin level <11.5 g/dL in children 5 to 11 years of age and hemoglobin level <12 g/dL in children 12 to 14 years of age.

### 2.4. Serum 25(OH)D concentrations and ascertainment of vitamin D status

Blood samples were collected during the examination, centrifuged, divided, and immediately frozen at–70°C on-site. Subsequently, the frozen samples were shipped on dry ice to a central laboratory for storage at–70°C until analysis. After acetonitrile-based extraction, serum 25(OH)D levels were measured at the National Center for Environmental Health (Atlanta, GA) using a radioimmunoassay kit (DiaSorin, Stillwater, MN). However, due to concerns about bias and imprecision of the DiaSorin radioimmunoassay, the centers for disease control and prevention developed regression equations to convert radioimmunoassay values to LC-MS/MS equivalents for NHANES 2001–2006. Furthermore, the centers for disease control and prevention laboratory analyzed serum 25(OH)D metabolites from 2007 to 2018 using LC-MS/MS. Total serum 25(OH)D (nmol/L) was calculated as the sum of 25(OH)D3 and 25(OH)D2, excluding the C3-epi-25(OH)D3 metabolite. Detailed information and procedures can be found on the NHANES website (https://wwwn.cdc.gov/ nchs/nhanes/vitamind/ analyticalnote.aspx).^[18]^ 25(OH)D values of <50 and <75 nmol/L defined the thresholds of deficiency and insufficiency, respectively. 25(OH)D values of ≥75 nmol/L defined the thresholds of sufficiency.

### 2.5. Magnesium intake

Dietary magnesium intake data for a 24-hour period was collected via a recall interview (midnight to midnight) conducted in mobile examination centers. This method, known as the 24-hour recall interview, is commonly used in large-scale surveys like NHANES to determine dietary intake based on expert consensus.^[19]^

The magnesium intake was categorized into 2 groups, based on Zweite value (208.5 mg/d) in the study population.

### 2.6. Other covariates

Our study considered age, gender, race/ethnicity, family income, PIR, BMI, blood lead, dietary intake such as total calories, iron, zinc, protein, niacin, vitamin B6, folate, vitamin B12, and fiber as covariates. In this study we categorized race/ethnicity (as defined by NHANES) as non-Hispanic White, other Hispanic, non-Hispanic Black, Mexican American, and other races. Family income was defined by the poverty income ratio. The dietary data were additionally collected for total calories, iron, zinc, protein, niacin, vitamin B6, folate, vitamin B12, and fiber.

Venous blood samples were collected to measure the blood lead level. The samples were diluted, stored at ‐20°C, and then analyzed using an Inductively Coupled Plasma Dynamic Reaction Cell Mass Spectrometer (ELAN DRC II, PerkinElmer, Norwalk) in the central laboratory, following the standard protocol.

### 2.7. Statistical analysis

Statistical software packages R (http://www.R-project.org, The R Foundation), Free Statistics software version 1.8 was used for data analysis. The sample weights were provided by NHANES (NCHS, 2005–2018). Continuous variables are presented as mean and standard deviation, categorical variables as numbers (n) and percentages (%), and skewed data as median and interquartile range. The population characteristics of the magnesium intake <208.5 mg/d group and ≥208.5 mg/d group were compared by performing 2-tailed Student *t* tests and chi-square tests. Logistic regression analysis was used for evaluating the risk of anemia from vitamin D deficiency and 25(OH)D concentration. Crude model was adjusted for no covariates and model 1 for sex, age, race, PIR, BMI. In model 2, blood lead, dietary intake including total calories, iron, zinc, protein, niacin, vitamin B6, folate, vitamin B12, and fiber were considered, in addition to model 1 adjustments. The subgroup analyses were performed between the vitamin D status groups including vitamin D deficiency, insufficiency, sufficiency group. The likelihood ratio test was employed to assess subgroup interaction. Odds ratios (ORs) and 95% confidence intervals (CIs) were calculated, with a 2-sided *P* < .05 indicating statistical significance.

## 3. Results

### 3.1. Baseline characteristics of the study population

**Figure 1.**
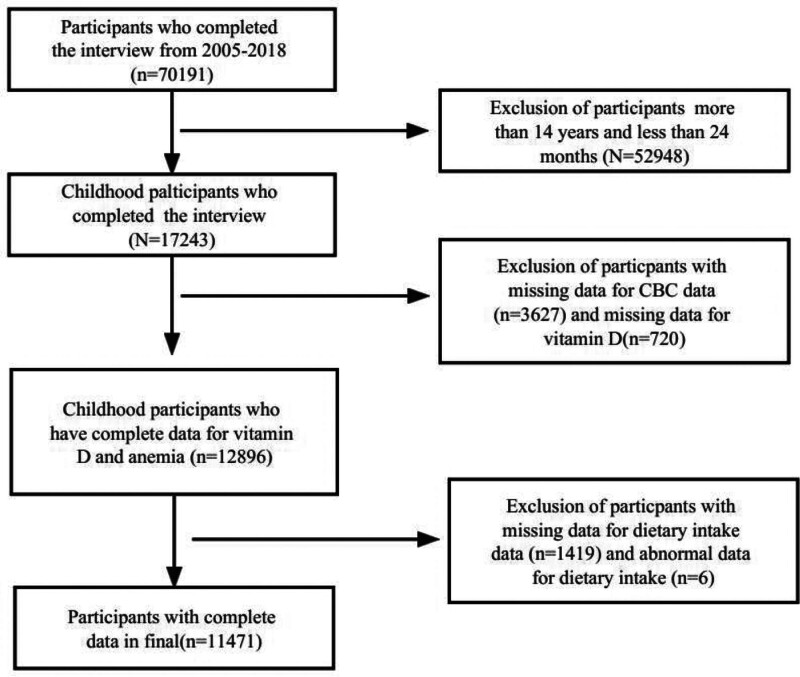
Flowchart of the study.

The flow chart of the exclusion criteria for the study population is shown in Figure 1. Of 11,471 US children eligible for our final analysis, 2370 (20.7%) 911 individuals defined as vitamin D deficiency. The overall mean age was 8.2 ± 3.5 years, 49.4% were female, and 3.8% had anemia. At baseline, characteristics between the Magnesium intake subgroups differed for almost all covariates. The prevalence of anemia in the magnesium intake <208.5 mg/d group and ≥208.5 mg/d group were 4.4% and 3.1%, respectively (Table 1).

**Table 1:** Baseline characteristics of the study sample.

Variables	Total (n = 11,471)	Magnesium intake		*P*-value
		<208.5 mg/d (n = 5716)	≥208.5 mg/d (n = 5755)	
Gender, n (%)				<.001
Male	5805 (50.6)	2598 (45.5)	3207 (55.7)	
Female	5666 (49.4)	3118 (54.5)	2548 (44.3)	
Age (yr)	8.2 ± 3.5	7.7 ± 3.6	8.7 ± 3.3	<.001
Race/ethnicity, n (%)				<.001
Mexican American	2965 (25.8)	1398 (24.5)	1567 (27.2)	
Other Hispanic	1176 (10.3)	548 (9.6)	628 (10.9)	
Non-Hispanic White	3228 (28.1)	1560 (27.3)	1668 (29)	
Non-Hispanic Black	2837 (24.7)	1601 (28)	1236 (21.5)	
Other race	1265 (11.0)	609 (10.7)	656 (11.4)	
BMI (kg/m^2^)	18.8 ± 4.5	18.6 ± 4.5	19.0 ± 4.5	<.001
PIR, median (IQR)	1.4 (0.8, 2.9)	1.4 (0.7, 2.8)	1.5 (0.8, 3.0)	<.001
Dietary factors				
kcal, median (IQR)	1715.0 (1365.5, 2114.0)	1398.2 (1152.4, 1669.0)	2062.0 (1761.5, 2433.0)	<.001
Protein (g)	63.8 ± 24.4	49.3 ± 15.3	78.1 ± 23.2	<.001
Fiber (g)	13.2 ± 6.0	9.5 ± 3.3	16.9 ± 5.8	<.001
Magnesium (mg)	219.2 ± 80.9	158.9 ± 34.1	279.1 ± 68.6	<.001
Iron(mg)	13.6 ± 6.2	10.6 ± 4.3	16.6 ± 6.4	<.001
Zinc(mg)	9.6 ± 4.5	7.3 ± 2.8	11.9 ± 4.6	<.001
Niacin (mg)	19.8 ± 8.3	15.7 ± 6.0	23.8 ± 8.4	<.001
Vitamin B6 (mcg), median (IQR)	1.5 (1.1, 2.0)	1.2 (0.9, 1.5)	1.9 (1.5, 2.4)	<.001
Folate (mcg), median (IQR)	325.5 (237.5, 438.0)	256.2 (191.5, 335.0)	403.5 (316.5, 518.8)	<.001
Vitamin B12 (mcg), median (IQR)	4.2 (2.9, 5.9)	3.4 (2.3, 4.6)	5.3 (3.9, 7.0)	<.001
Laboratory factors				
25(OH)D (nmol/L)	64.9 ± 18.8	64.3 ± 19.0	65.6 ± 18.5	<.001
25(OH)D subgroup, n (%)				<.001
<50 nmol/L	2370 (20.7)	1289 (22.6)	1081 (18.8)	
50–75 nmol/L	5985 (52.2)	2906 (50.8)	3079 (53.5)	
≥75 nmol/L	3116 (27.2)	1521 (26.6)	1595 (27.7)	
Blood lead (µg/dL) median (IQR)	0.8 (0.5, 1.3)	0.9 (0.6, 1.3)	0.8 (0.5, 1.2)	<.001
Anemia, n (%)				<.001
No	11,038 (96.2)	5464 (95.6)	5574 (96.9)	
Yes	433 (3.8)	252 (4.4)	181 (3.1)	

BMI = body mass index, PIR = poverty income ratio.

### 3.2. Association of covariates and anemia risk in children

From the univariate analysis results, it was revealed that gender, race/ethnicity, family income, BMI, some dietary indices such as total calories, iron, zinc, protein, niacin, vitamin B6, folate, vitamin B12, fiber, and magnesium were associated with anemia (Table 2).

**Table 2:** Association of covariates and anemia risk.

Variable	OR_95 CI	*P*-value
Age	0.985 (0.958–1.012)	.2719
Gender, n (%)		
Male	1 (Ref)	
Female	1.214 (1.001–1.471)	.0489
Race/ethnicity, n (%)		
Mexican American	1 (Ref)	
Other Hispanic	1.547 (1.004–2.381)	.0477
Non-Hispanic White	0.635 (0.421–0.959)	.0308
Non-Hispanic Black	5.02 (3.739–6.74)	<.001
Other race	2.316 (1.584–3.386)	<.001
PIR	0.824 (0.765–0.887)	<.001
BMI	1.03 (1.011–1.05)	.0021
Dietary factors		
kcal	1 (0.999–1)	1.00E‐04
Protein	0.994 (0.99–0.999)	.0084
Fiber	0.964 (0.947–0.982)	1.00E-04
Niacin	0.98 (0.968–0.993)	.0018
Vitamin B6	0.832 (0.724–0.956)	.0095
Folate	0.999 (0.998–0.999)	1.00E-04
Vitamin B12	0.942 (0.904–0.982)	.0051
Magnesium	0.997 (0.996–0.998)	<.001
Iron	0.972 (0.955–0.989)	.0011
Zinc	0.959 (0.936–0.983)	9.00E-04
Laboratory factors		
25(OH)D	0.974 (0.969–0.98)	<.001
25(OH)D subgroup		
<50 nmol/L	1 (Ref)	
50–75 nmol/L	0.524 (0.423–0.65)	<.001
≥75 nmol/L	0.338 (0.254–0.45)	<.001
Serum lead	0.946 (0.85–1.052)	.3021

BMI = body mass index, PIR = poverty income ratio.

### 3.3. Associations between 25(OH)D and anemia risk

Table 3 shows that when compared to the vitamin D sufficiency group, vitamin D sufficiency participants had a higher OR (OR = 2.96, 95% CI: 2.222–3.943) for anemia risk in crude model. When adjusted for sex, age, race/ethnicity, PIR and BMI in model 1, the OR was 1.48 (95% CI: 1.069–2.049). Further, based on model 1 and additionally adjusted for blood lead, dietary intake including total calories, iron, zinc, protein, niacin, vitamin B6, folate, vitamin B12, and fiber in model 2, the OR was 1.492 (95% CI:1.072–2.077). Among associations between 25(OH)D concentration and anemia risk, we found that 25(OH)D concentration was negatively associated with anemia risk (OR = 0.974, 95% CI: 0.969–0.98, *P* < .001). In adjusting models 1 and 2, similar results were obtained. Figure S1, Supplemental Digital Content, https://links.lww.com/MD/Q788 shows hemoglobin level disparities among various age groups and vitamin D statuses.

**Table 3:** The associations between serum 25(OH)D and anemia risk in different models.

25(OH)D (nmol/L)	Participants/cases	Crude model		Model 1		Model 2	
		OR (95% CI)	*P*-value	OR (95% CI)	*P*-value	OR (95% CI)	*P*-value
25(OH)D	11,471/433	0.974 (0.969–0.98)	<.001	0.988 (0.981–0.994)	2.00E‐04	0.988 (0.981–0.994)	2.00E‐04
Subgroup							
VDS	3116/71	1 (Ref)		1 (Ref)		1 (Ref)	
VDD	2370/153	2.96 (2.222–3.943)	<.001	1.48 (1.069–2.049)	.0183	1.492 (1.072–2.077)	.0176
VDI	5985/209	1.552 (1.181–2.039)	0.0016	1.101 (0.826–1.467)	.5124	1.121 (0.839–1.496)	.4401
*P*-trend		0.57 (0.495–0.657)	<.001	0.807 (0.687–0.948)	.0091	0.806 (0.684–0.949)	.0098

Data presented are ORs and 95%Cis. Model 1: adjusted for sex, age, race, PIR, BMI; model 2: adjusted for all covariates listed in Table 1.

25(OH)D <50 nmol/L defined as vitamin D deficiency (VDD), 25(OH)D 50–75 nmol/L defined as vitamin D insufficiency (VDI), 25(OH)D ≥ 75 nmol/L defined as vitamin D sufficiency (VDS).

### 3.4. Dietary magnesium intake ameliorates the association between vitamin D deficiency and risk of anemia in children

An interaction was found between magnesium intake and vitamin D deficiency with respect to anemia risk (interaction likelihood ratio test: *P* = .009) (Table 4). Stratified analysis by magnesium intake was performed. In 1 group (high magnesium intake: ≥208.5 mg/d), these rates of anemia risk in the vitamin D deficiency group, insufficiency group and sufficiency group were 4%, 3.2%, and 2.4%, respectively. Adjusting for other confounders, the results of the multivariable logistic regression showed that there was no positive association between vitamin D deficiency and anemia risk (OR = 0.934, 95% CI: 0.564–1.548, *P* = .7916). However, in the other group (low magnesium intake: <208.5 mg/d), The rate of anemia risk in the vitamin D deficiency group, insufficiency group and sufficiency group were 8.5%, 3.8%, and 2.2%, respectively. When adjusting for other confounders in the populations with a low magnesium diet, vitamin D deficiency increased the risk of anemia by 99.2% compared with vitamin D sufficiency group (OR = 1.992, 95% CI: 1.264–3.139, *P* = .003). An interaction was also found between magnesium intake and 25(OH)D concentration with respect to anemia risk (interaction likelihood ratio test: *P* = .007) (Table 5).

**Table 4:** Association between serum vitamin D and anemia risk by dietary magnesium intake.

Subgroup	N (%)	OR	95% CI	*P*-value	*P* for interaction
Magnesium intake <208.5 mg/d (n = 5716)					
Vitamin D sufficiency	33 (2.2)	1(Ref)			.009
Vitamin D deficiency	110 (8.5)	1.992	(1.264–3.139)	.003	
Vitamin D insufficiency	109 (3.8)	1.203	(0.794–1.824)	.3824	
Magnesium intake ≥208.5 mg/d (n = 5755)					
Vitamin D sufficiency	38 (2.4)	1(Ref)			
Vitamin D deficiency	43 (4)	0.934	(0.564–1.548)	.7916	
Vitamin D insufficiency	100 (3.2)	1.038	(0.692–1.558)	.8565	

Data presented are ORs and 95% CIs. Adjusted for all covariates listed in Table 1.

25(OH)D <50 nmol/L defined as vitamin D deficiency, 25(OH)D 50–75 nmol/L defined as vitamin D insufficiency, 25(OH)D ≥75 nmol/L defined as vitamin D sufficiency.

**Table 5:** Association between serum 25(OH)D and anemia risk by dietary magnesium intake.

Subgroup	N (%)	OR	95% CI	*P*-value	*P* for interaction
Magnesium intake <208.5 mg/d (n = 5716)					
25(OH)D	252 (4.4)	0.982	(0.974–0.991)	.0001	.007
Magnesium intake ≥208.5 mg/d (n = 5755)					
25(OH)D	181 (3.1)	0.996	(0.987–1.006)	.4706	

Data presented are ORs and 95% CIs. Adjusted for all covariates listed in Table 1.

## 4. Discussion

This study, which used a nationally representative sample of the general US population, discovered a significant association between vitamin D deficiency and increased risk of anemia in children. Furthermore, we have reported the interaction between daily intake of magnesium and vitamin D deficiency with respect to anemia risk in children. This suggests that dietary magnesium intake may ameliorate the detrimental effect of vitamin D deficiency on anemia risk in children.

Our study supports previous research in children, confirming that vitamin D deficiency increases the risk of anemia. We utilized NHANES database from 2005 to 2018, while literature^[20]^ used database from 2001 to 2006, yet both studies obtained consistent effect values: 1.492 for us and 1.47 for literature.^[20]^ The prevalence of anemia was 2.15 times higher in the vitamin D deficiency group in our study, compared to the adequacy group, whereas literature^[5]^ reported a 3.45 times higher prevalence. To address age-related variations in anemia diagnostic criteria, we analyzed the impact of vitamin D status on hemoglobin levels within WHO-defined age groups. Our findings revealed significantly lower hemoglobin levels in the vitamin D deficient group at all ages, reinforcing the reliability of the results. Although our study excluded children under 2 years of age, relevant literature sources,^[20–23]^ that included this age group reached consistent conclusions. In summary, comprehensive data from our study and the existing literature firmly support an increased risk of vitamin D deficiency and anemia in children from 0.3 months up to 14 years of age.

Inconsistencies with our findings primarily pertain to the infant and young child population, which constitutes a distinct subset throughout childhood. In infancy specifically, multiple factors including maternal and gestational health status, physiological anemia, and feeding status exert notable influence on the occurrence of anemia. One study in Jordan^[7]^ focused on this specific population, examining the relationship between vitamin D deficiency and anemia in 203 children aged 6 to 36 months receiving primary care. Their findings revealed an overall anemia prevalence of 40.0%, with rates of 51.8% among infants and 26.9% among toddlers. However, no significant association was observed between vitamin D status and anemia in their analysis. Similarly, an investigation conducted in India^[8]^ explored the correlations between 6 nutrients and anemia risk in infants and toddlers, highlighting only an association between iron intake and anemia within this age group, while no correlation between vitamin D deficiency and anemia was found. These consistent outcomes from these both studies suggest the necessity for additional data to comprehensively examine the association between vitamin D deficiency and anemia in infants and toddlers. It is plausible that the risk of anemia respect to vitamin D deficiency in this age group differs from that in other age groups, warranting independent analysis.

Magnesium, as a cofactor for over 600 enzymes, is essential for numerous biochemical reactions.^[24]^ Similarly, magnesium also serves as a coenzyme for enzymes involved in vitamin D metabolism and function, specifically influencing the activity of crucial rate-limiting enzymes including 1α-hydroxylase and 24-hydroxylase.^[14,15]^ Its presence facilitates the activation of these enzymes, thereby promoting efficient vitamin D metabolism and functionality. Consequently, maintaining adequate magnesium levels is vital for proper vitamin D metabolism. The findings of our study demonstrate that low magnesium intake significantly amplifies the risk of anemia associated with vitamin D deficiency by 99.2%. Conversely, in individuals with high magnesium intake group, vitamin D deficiency does not heighten the risk of anemia. This result indirectly highlights the contribution of magnesium ions to pivotal role of vitamin D in the hematopoietic system, thus providing clinical evidence supporting magnesium’s significance in vitamin D metabolism and function. Despite these insights, there remains a scarcity of similar studies conducted at the clinical level. Therefore, further investigation using additional clinical data is necessary to confirm these conclusions.

Our study had some limitations. First, cross-sectional studies lack the capacity to establish a temporal cause–effect relationship due to their inherent nature. Second, self-reported data collected through mobile examination center interviews may be subject to recall bias. Moreover, multiple 24-hour dietary recalls cannot accurately reflect long-term magnesium status. Third, biochemical parameters, such as serum magnesium, and the type of anemia information were unavailable in NHANES database. Fourth, despite employing the multistage stratified probability design method, the participants in the NHANES database are primarily American citizens and may not be fully representative of individuals residing in other regions globally.

Finally, our study was susceptible to unmeasured confounding variables. Considering these limitations, well-designed multicenter controlled trials may be necessary to validate our findings.

## 5. Conclusions

There appears to be a synergistic relationship between vitamin D and magnesium ions in the prevention of anemia. Optimal activation and functionality of vitamin D appears to be dependent on sufficient levels of magnesium. Consequently, emphasizing intake of both vitamin D and magnesium may yield greater benefits in the prevention of anemia among children.

## Acknowledgments

We are thankful to J. Liu (People’s Liberation Army of China General Hospital, Beijing, China) for his help in this revision. Dr Liu has given permission to be acknowledged in this publication.

## Author contributions

**Data analysis and writing – original draft:** Wenxin Gu.

**Critical feedback and revision suggestions on the study design:** Yue Wang, Chunhuai Li.

**Data collection:** Shuangshuang Wu, Shuangshuang Liu, Chun Chang.

**Study design and writing – review & editing:** Lu Xue.

## Supplementary Material
