# Supplementary material for: Dietary magnesium intake as modifier of the association between vitamin D deficiency and risk of anemia in the US children aged 2–14 years: A cross-sectional study
Source: Medicine (Baltimore). 2025 Dec 12;104(50):e46264. doi: 10.1097/MD.0000000000046264 (PMC12708146; doi:10.1097/MD.0000000000046264)
Supplement: Supplementary file 1 [file medi-104-e46264-s001.pdf]

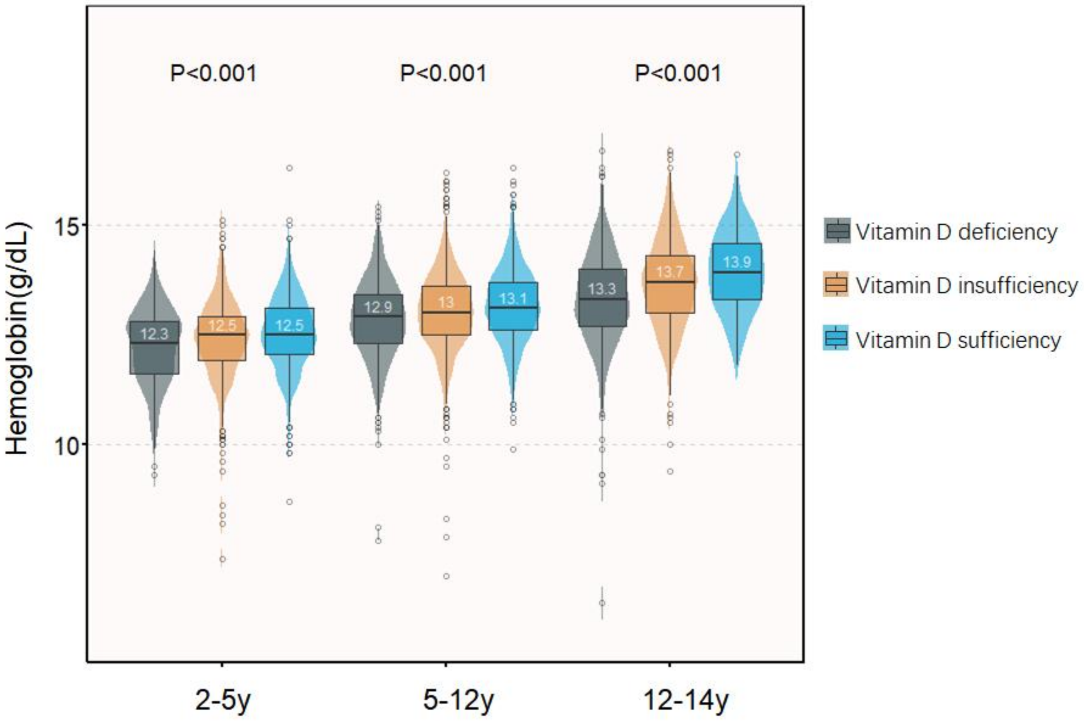

Supplementary Fig 1 Hemoglobin level disparities among various agegroups and vitamin D statuses.Age groups were stratified based on WHO diagnostic criteria for anemia. Comparative analysis was conducted to assess the variations in hemoglobin levels across different age groups and vitamin D statuses. The results revealed significant differences ($p < 0.001$) in hemoglobin levels among the three age groups and across the various vitamin D statuses.
